# Differences in disease phenotype and severity in SLE across age groups

**DOI:** 10.1177/0961203316644333

**Published:** 2016-05-04

**Authors:** N Ambrose, T A Morgan, J Galloway, Y Ionnoau, M W Beresford, D A Isenberg

**Affiliations:** 1 Centre for Rheumatology, University College London (UCL) Hospital NHS Foundation Trust, London, UK; 2 Arthritis Research UK Centre for Adolescent Rheumatology, UCL, UK; 3 Department of Paediatric Rheumatology, Alder Hey Children’s NHS Foundation Trust, Liverpool, UK; 4 Department of Women’s and Children’s Health, Institute of Translational Medicine, University of Liverpool, UK

**Keywords:** Cohort study, juvenile-onset SLE, childhood-onset SLE

## Abstract

**Objectives:**

Significant differences have been reported in disease phenotype and severity of systemic lupus erythematosus (SLE) presenting in different age groups. Most indicate a more severe phenotype in juvenile-onset SLE (JSLE). There have been limited studies in older patients and no large studies looking at SLE across all age groups.

**Methods:**

We assessed the effect of age of onset of SLE on the clinical phenotype by analysing data from two large UK cohorts (the UK JSLE Cohort and the UCLH SLE cohort).

**Results:**

A total of 924 individuals were compared (413 JSLE, 511 adult-onset SLE). A female preponderance was present, but less pronounced at either end of the age spectrum. Arthritis was more common with advancing age (93% vs 72%, *p* < 0.001), whereas renal disease (44% vs 33%, *p* = 0.001), alopecia (47% vs 23%, *p* < 0.001) and aphthous ulcerations (39% vs 26%, *p* = 0.001) were more common in the young. Neuropsychiatric lupus was less common in mature-onset SLE (*p* < 0.01). JSLE was associated more commonly with thrombocytopenia (21% vs 15%, *p* = 0.01), haemolytic anaemia (20% vs 3%, *p* < 0.001), high anti-dsDNA (71% vs 63%, *p* = 0.009), Sm (22% vs 16%, *p* = 0.02) and RNP (36% vs 29%, *p* < 0.04) auto-antibodies. Leucopenia increased with advancing age (*p* < 0.001). Mortality has been declining over recent decades. However, death rates were substantially higher than the general population. The standardized mortality ratio was 18.3 in JSLE and 3.1 in adult-onset SLE.

**Conclusion:**

These data from the largest-ever direct comparison of JSLE with adult-onset SLE suggest an aggressive phenotype of disease with a worse outcome in patients with JSLE and emphasizes the importance of careful follow-up in this population.

## Background

Systemic lupus erythematosus (SLE) is a severe, complex, multi-system autoimmune rheumatic disease. It is most prevalent among women of childbearing age.^1–3^ Approximately 20% of cases begin during childhood, usually after puberty,^4–6^ and published data suggest a further 10–20% present after the age of 50 years.^7–9^

Summaries of the main clinical and laboratory features of juvenile-onset SLE (JSLE) have been reported by several groups including our own,^10^ with most reports finding a severe phenotype with a greater burden of steroids and immunosuppressive treatment.^10–15^ However, previous comparison studies generally assessed fewer than 100 JSLE cases. Additionally few, if any, studies have looked at SLE across the broader age groups within the same study.

Previous studies have provided conflicting results regarding the changes in clinical phenotype across age groups. Most studies find more renal disease and more haematological involvement in JSLE. However, for many other features including mortality risk, neuropsychiatric lupus (NPSLE), serositis, arthritis and autoantibody profiles, reports are conflicting. The reasons for the disparity are likely to be multifactorial, with relatively small cohort size, retrospective data collection and/or variability in case definitions contributing to the differences.

In this report we describe the effect of age at onset of SLE on the phenotypic manifestations by assessing two large cohorts: the United Kingdom (UK) JSLE Cohort Study and the University College London Hospital (UCLH) cohort.

## Methods

We combined two cumulative historical databases to assess clinical and laboratory features and how they related to age at onset of SLE. All patients fulfilled the revised criteria for the classification of SLE as set out by the American College of Rheumatology (ACR).^16,17^

### UK JSLE Cohort

A national UK JSLE observational cohort study was commenced in 2006, and to date over 400 patients have been enrolled. Almost all UK centres (*n* = 21) treating patients diagnosed with JSLE participate. Detailed clinical phenotypic data are obtained at baseline. Patients are included who are diagnosed prior to the age of 17 years old. The entire cohort includes patients meeting two or more ACR classification criteria, but for the purpose of this report, we have included only those who have accrued four or more ACR criteria by the time of latest follow-up.^16,17^ Along with baseline and annual visits, phenotypic data are collected at the time of patients’ routine clinical follow-up. Data are collected prospectively in a central database at the University of Liverpool.

### UCLH SLE juvenile and adult cohort

UCLH is a large multi-racial UK tertiary referral centre for SLE. A common database established from patient interviews and chart reviews was created and data have been prospectively entered once four or more ACR criteria were present. Information from 636 patients in this cohort was included in this study. A third of patients are from the hospital’s catchment area, a third from Greater London and a third from the wider UK.

### Combination of two large cohorts

The authors had access to both databases (MWB and DI, respectively). As it was possible for patients to be enrolled in both studies, all duplicates were removed at the point of merging. A core dataset reflecting variables collected across both cohorts was created. A total of 924 individuals diagnosed with SLE and meeting ≥4 ACR criteria were included once duplicates had been removed. As well as disaggregated ACR criteria, cardiovascular events (any event deemed by the treating physician to be a cardiovascular event such as a myocardial infarct) and all-cause mortality data were compared between cohorts.

Information regarding patients that had been collected on both databases (*n* = 24) was used as a tool to validate recorded information between the cohorts.

The following differences between the cohorts were noted: (1) UK JSLE study median follow-up was 3.7 years; UCLH median follow-up was 15 years. (2) NPSLE was defined as per ACR guidelines^18^ but headaches were not included in the UCLH cohort as it was deemed that they were present almost universally over long-term follow-up of such a group; therefore headaches were excluded.^3^ Ethnicity recording varied across cohorts, reflecting in part, changing terminology over time. Broad classifications of White, Black, Asian (including South Asian and Chinese) or Other were adopted.

### Age definitions

JSLE was defined for this combined study as SLE with onset before the patient’s 18th birthday. This group was further subdivided. Childhood onset was defined as onset before the 12th birthday and adolescent onset was defined as those patients diagnosed between the ages of 12 and 17 years. Adult-onset SLE was defined as those patients 18 years or older at the time of diagnosis. This group was further subdivided. Adults of 18–49 years at time of diagnosis were described as adult onset. Patients 50 years or older at time of onset were defined as the mature-onset group.

### Serology

Serological results were recorded as follow: the presence or absence of persistent hypocomplementemia (C3); antinuclear antibody (ANA) positivity (measured with Hep2 cells with a cut-off of > 1:80); anti-double-stranded DNA (anti-dsDNA) antibody positivity (over twice the upper limit of normal by commercial enzyme-linked immunosorbent assay (ELISA) or a positive *crithidia*, on two occasions), anti-extractable nuclear antigen (anti-ENA) positivity (Ro, La, Sm, and ribonuclear proteins (RNP), by commercial ELISA). All laboratory tests were performed in the patient’s local centre as part of routine clinical care.

### Data analysis

Data were analysed by univariable and multivariable analysis for demographic, renal biopsy, serological and clinical data. Significance testing between groups was performed by Chi square testing across patient characteristics. Mortality rates were calculated using direct standardization methods. Individual year population mortality data from 1901 until 2013 were retrieved from the Office for National Statistics Summary Reports.^19^ Age, gender and calendar year standardized mortality ratios (SMRs) were calculated with a 90% confidence interval (CI) for the cohorts. Mortality rates for each decade (based upon year of diagnosis) were calculated, and Cuzick’s test for trend was used to determine significance. All analyses were conducted in Stata (v14).

## Results

A total of 924 individuals diagnosed with SLE and meeting ≥4 ACR revised criteria were compared, comprising 413 JSLE (0–17 years at diagnosis) and 511 adult-onset SLE patients (≥18 years).

### Demographics

Demographics of the cohort are shown in Table 1. A female preponderance was present but less extreme at either end of the age spectrum. There were significantly more male patients (17% vs 8%; *p* < 0.01) in the JSLE population compared with the adult population. Overall the ratio of female to males (F:M) was 6:1 in JSLE, 5:1 in younger ages, and 7:1 in older children. Adults had a ratio of 13:1 and 11:1 in the mature-onset group. It was observed that although the overall F:M ratio of the mature-onset SLE group remained high at 11:1; the ratio for patients diagnosed from the age of 60 dropped to 3:1. Older patents were more likely to be White, and higher percentages of patients of Asian ethnicity were found within the JSLE population (*p* < 0.01).

**Table 1:** Demographic data of the whole SLE cohort

Characteristic	**All JSLE**	Childhood	Adolescent	**All adult**	Adult	Mature
Numbers	**413**	136	277	**511**	467	44
Female:Male	**6:1**	5:1	7:1	**13:1**	13:1	11:1
Median age	**13**	10	14	**31**	30	54
Ethnicity (%)						
White	**50**	45	53	**60**	58	84
Asian	**25**	31	28	**10**	14	4
Black	**25**	21	17	**25**	26	9

Patients grouped by age of diagnosis: All JSLE ≤ 18 years; Childhood ≤ 12 years; Adolescent = 12–17 years; All adult = 18 years or older; Adult = 18–48 years; Mature = 50 years or older. SLE: systemic lupus erythematosus; JSLE: juvenile systemic lupus erythematosus.

### Clinical manifestations at different ages

Table 2 presents the ACR clinical characteristics (non-renal, non-NPSLE manifestations) between cohorts. No significant differences were observed in percentages of patients with a lupus rash between any groups. Rashes were common both in juvenile and adult lupus. There was also no difference in prevalence of photosensitivity. JSLE patients were more likely to report alopecia with a clear reduction of patients reporting alopecia with advancing age. JSLE patients also reported higher rates of oral ulcers. Adults were significantly more likely to report arthritis and serositis. There was a non-statistically significant reduction in serositis in the mature group.

**Table 2:** General clinical characteristics of the whole SLE cohort (% of whole SLE cohort)

Characteristic	**All JSLE**	Childhood	Adolescent	**All adult**	Adult	Mature	**Significance (*p*)**
Rash	**60**	70	70	**67**	68	52	**0.362**
Photosensitivity	**34**	33	35	**41**	42	30	**0.057**
Alopecia	**47**	48	46	**23**	24	18	**<0.001**
Oral ulcers	**39**	35	41	**26**	26	32	**<0.001**
Joint	**72**	66	75	**93**	93	98	**<0.001**
Serositis	**23**	18	26	**41**	43	27	**<0.001**

Data displayed as percentages of the whole group. Patients grouped by age of diagnosis: All JSLE ≤ 18 years; Childhood ≤ 12 years; Adolescent = 12–17 years; All adult = 18 years or older; Adult = 18–48 years; Mature = 50 years or older. Significance (*p*) = comparison of All JSLE to All adult SLE patients. SLE: systemic lupus erythematosus; JSLE: juvenile systemic lupus erythematosus.

### Renal manifestations

**Figure 1:**
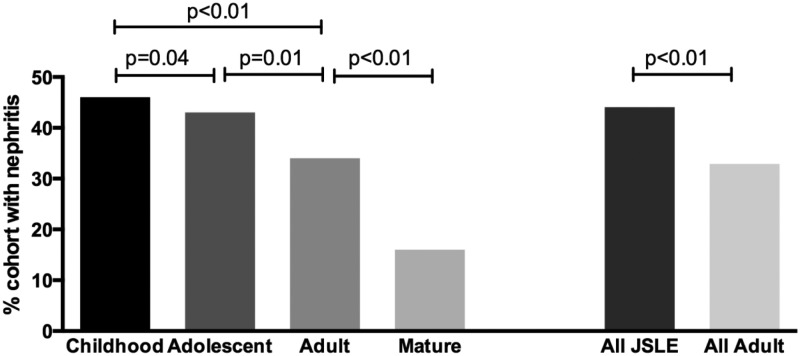
Breakdown of renal involvement by age. The left-hand bars show the breakdown by age as divided into four groups. The right-hand bars summarize key differences between JSLE and adult SLE groups. JSLE: juvenile systemic lupus erythematosus; SLE: systemic lupus erythematosus.

JSLE was associated with a significantly higher prevalence of lupus nephritis (44% vs 33%, *p* = 0.001) (Figure 1). Thirty-one per cent of JSLE patients had biopsy-proven lupus nephritis compared with 27% of adult-onset SLE patients. In these patients the median disease duration at the time of first renal biopsy was two months in JSLE patients and 24 months in adult-onset SLE. There was no difference in subtypes of renal nephritis, with diffuse proliferative glomerulonephritis the most common subtype in both groups.

### NPSLE

**Figure 2:**
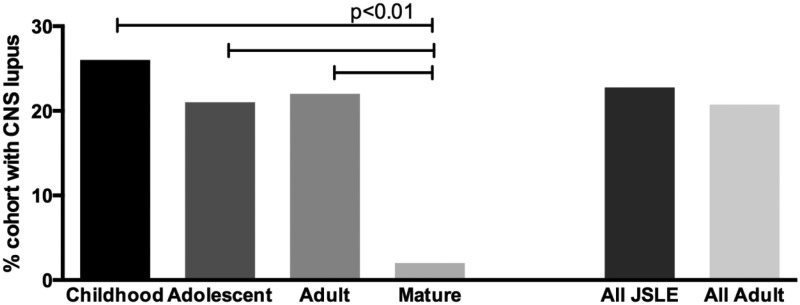
Breakdown of CNS lupus by age. The left-hand bars show the breakdown by age as divided into four groups. The right-hand bars summarize key differences between JSLE and all-adult SLE groups. CNS: central nervous system; JSLE: juvenile systemic lupus erythematosus; SLE: systemic lupus erythematosus.

Mature-onset SLE patients were far less likely to have NPSLE than any other group (Figure 2). We did not find a statistically significant difference between JSLE and adult SLE NP involvement but there was a trend towards the JSLE cohort having more neurological disease.

### Haematological and immunological manifestations

Table 3 shows the laboratory characteristics across the cohorts. Laboratory results were available for all participants. Lymphopenia was more prevalent with increasing age. Thrombocytopenia and haemolytic anaemia were both significantly more prevalent in the JSLE group. Overall the JSLE group was significantly more likely to have ‘ACR haematological involvement’ than adult-onset disease.

**Table 3:** Haematological and immunological involvement (% of the whole SLE cohort)

	**All JSLE**	Childhood	Adolescent	**All adult**	Adult	Mature	**Significance (*p*)**
Haematological							
Lymphopenia	**62**	51	68	**76**	75	84	**<0.001**
Leucopenia	**32**	29	34	**30**	31	25	**0.590**
Thrombocytopenia	**21**	19	22	**15**	15	16	**0.011**
Haemolytic anaemia	**20**	24	19	**3**	3	0	**<0.001**
Coombs positive	**24**	24	23	**21**	21	16	**0.350**
ACR ‘haematological’							**0.046**
Immunological							
ANA	**97**	98	97	**94**	94	95	**0.061**
dsDNA	**71**	75	69	**63**	63	57	**0.009**
Low C3	**62**	57	65	**46**	48	23	**<0.001**
Sm	**22**	19	23	**16**	17	7	**0.021**
RNP	**36**	33	37	**29**	30	18	**0.038**
Ro	**35**	30	37	**38**	40	23	**0.335**
La	**17**	13	18	**15**	15	14	**0.450**
ACR ‘immunological’							**0.075**

Data displayed as percentages of the whole group. Patients grouped by age of diagnosis: All JSLE ≤ 18 years; Childhood ≤ 12 years; Adolescent = 12–17 years; All adult = 18 years or older; Adult = 18–48 years; Mature = 50 years or older. Significance (*p*) = comparison of All JSLE to All adult SLE patients. ACR ‘haematological’ = any haematological involvement by ACR criteria. ACR ‘immunological’: any immunological involvement by ACR criteria; SLE: systemic lupus erythematosus; JSLE: juvenile systemic lupus erythematosus; ACR: American College of Rheumatology; ANA: antinuclear antibodies; dsDNA: double-stranded DNA.

ANA was positive in over 94% of patients in all groups. The JSLE group had a significantly higher prevalence of immunological findings including positive anti-dsDNA antibodies, anti-RNP and anti-Sm autoantibodies. Low complement C3 was also more prevalent in the JSLE group.

### Cardiovascular events

In total, 12 cardiovascular events were recorded in JSLE patients (3%) with a median age at event of 16 years and median disease duration two years. In contrast, 47 events were recorded in the adult cohort (9%), with a median age at event 47 years and median disease duration 10 years.

### Mortality

**Figure 3:**
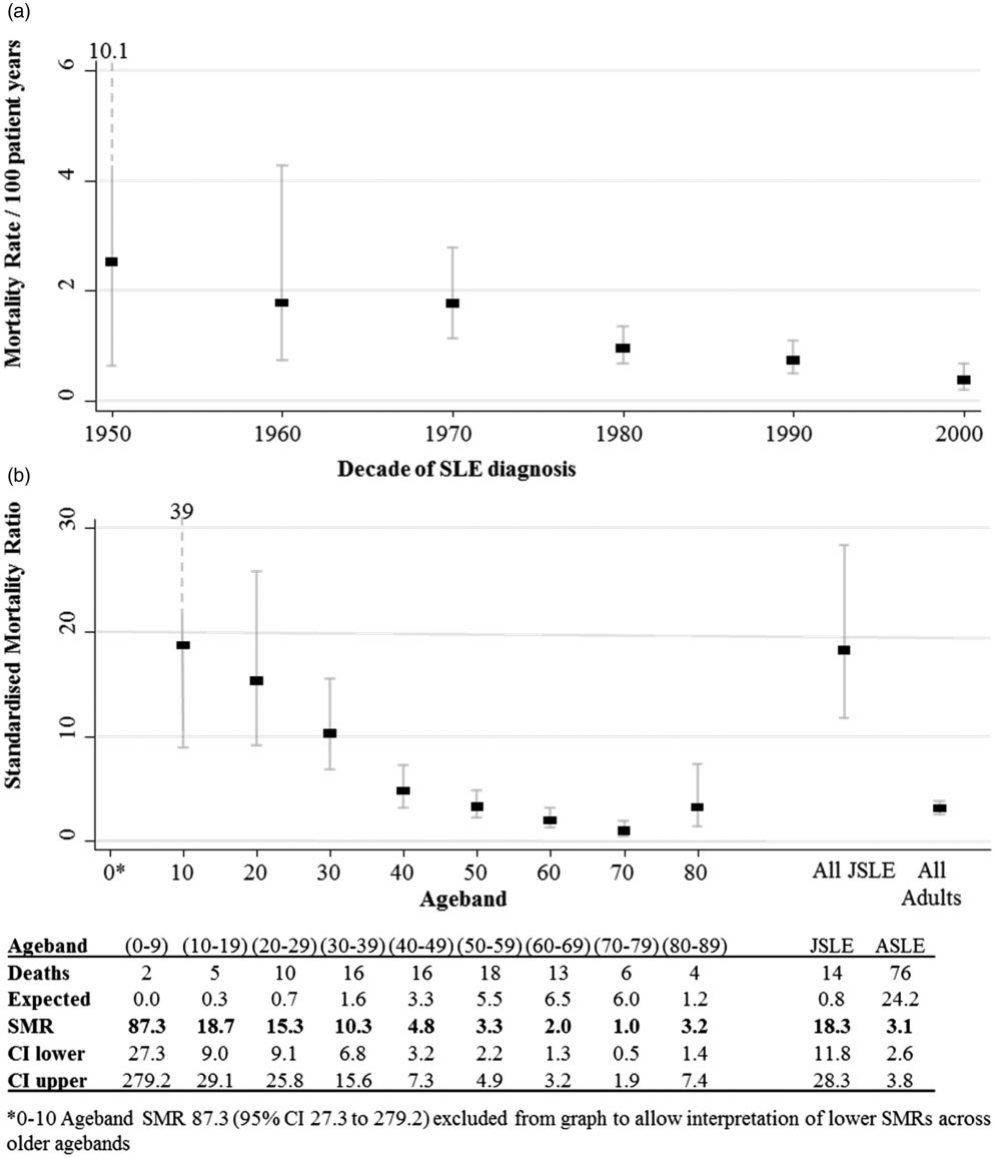
(a) Mortality rate expressed per 100 patient years, by decade of diagnosis of SLE, showing a reduction of mortality rates over time. (b) Standardized mortality ratio (SMR) of entire cohort by age, grouped into decades, with 90% confidence intervals. Deaths: deaths per 100 patient years; Expected: expected deaths per 100 in England and Wales population; Lower: 90% lower confidence interval; Upper: 90% upper confidence interval; SLE: systemic lupus erythematosus.

Of the 882 patients with sufficient long-term information (patients who had moved away or were lost to follow-up were excluded), 95 deaths occurred during follow-up. The overall mortality rate in the JSLE group was 0.5/100 patient years (pyrs) (95% CI 0.3 to 0.9) and in the adult SLE group was 10.7/100 pyrs (95% CI 0.8 to 13.5). Mortality rates by age of onset declined over the decades: 1950: 2.5/100 pyrs (CI 0.6 to 10.1); 2000: 0.4 (CI 0.2 to 0.7) (Figure 3(a)). The trend in changing mortality was statistically significant (*p* = 0.019).

When standardized to the expected mortality rates of the general population, the JSLE group was found to have an SMR of 18.3 (CI 11.8 to 28.3) and the adult SLE group 3.1 (CI 2.6 to 3.9). The age-specific mortality rates are shown in Figure 3(b). The SMR in the 0–9 age group was particularly elevated at 87 (CI 27 to 292); therefore, this group was excluded from the graphic (Figure 3(b)) to enable appreciation of the other SMRs. A non-statistically significant trend was seen towards higher mortality rates amongst the female population.

## Discussion

Comparison of SLE phenotype by age group in two large UK cohorts revealed similarities, but also important differences between the age groups. Previous studies have suggested disease to be more severe in the juvenile population, with a milder course in mature onset, and our results strongly support this.^4,11–13,15,20–26^ Importantly we identified that young age is a critical factor in SMR.

As with other studies we found a female predominance that was less pronounced at both ends of the age spectrum. In adults the female:male ratio was 13:1, which is higher than some groups report. Compared to other studies mostly based in North America,^27^ our data showed a low percentage of non-Whites. Older patents were even more likely to be White with higher percentages of patients of Asian ethnicity within the JSLE population. There are very few large studies specifically looking at mature-onset SLE. Available studies report an incidence of 12–20% of adult-onset SLE.^7–9,28–30^ Overall 5% of our patients were mature at onset, or 9% of the whole adult-onset population.

No difference was observed in the prevalence of a lupus rash between any groups. Others have reported that a malar rash may be more frequent in children^5,21,24,31,32^ and that a discoid rash is more common in adults.^5^ We did not differentiate between discoid and malar rash. JSLE was also associated with more frequently reported alopecia with a clear reduction of alopecia with advancing age. A meta-analysis of mature-onset SLE patients has also reported a lower prevalence of alopecia.^7^ We found that arthritis increased with advancing age, which is in agreement with some studies^8,15,22^ but not with others.^9,28,30,32^

Serositis was more common in adult-onset than juvenile-onset disease, although we did note a non-statistically significant drop-off of this finding in the mature-onset group. Some literature agrees^9,30^ with this observation, but other studies have found more serositis in the older age group.^8^ Further studies specifically addressing this with large cohorts of older patients are required.

JSLE was associated with more frequent lupus nephritis whereas nephritis was uncommon in mature-onset SLE. Other studies have consistently shown more frequent renal involvement in JSLE,^12,15,21,23,25,32^ and indeed groups have shown that JSLE is a risk factor for progression to dialysis.^33^ Studies looking at mature-onset SLE have found renal involvement to be less common in that age group.^9^

We found that mature-onset SLE patients were far less likely to have NPSLE disease than any other group. Previous studies including two meta-analyses of mature-onset SLE cohort data are in agreement with this.^7,9,32^ Interestingly, we did not find a statistical difference in prevalence between juvenile-onset and adult-onset NPSLE, despite previous reports suggesting NPSLE involvement may be more common in JSLE.^12,15,31^ There was a trend towards the JSLE cohort having more neurological disease. Other groups have subanalysed their data and have found JSLE to be associated with seizures,^22,24^ and one study reported that older age was associated with more peripheral neuropathies.^31^ Previous reports have used methods that have varied widely with some groups having narrow definitions of NPSLE as psychosis or seizures (ACR criteria) or others using the updated ACR criteria, which even include headaches. We had chosen not to classify headaches as NPSLE, in order to capture the more severe end of the neurological spectrum.

We found lymphopenia to be more prevalent with increasing age whereas JSLE was associated with thrombocytopenia and haemolytic anaemia. Haematological manifestations in JSLE have been described to include leucopenia, thrombocytopenia and haemolytic anaemia.^5,12,15,24,32^ The JSLE group had a higher prevalence of immunological involvement including anti-dsDNA, anti-RNP and anti-Sm autoantibodies. JSLE was also associated with low complement C3. These findings were in keeping with other studies.^15,20,32^ Autoantibodies were less common in mature-onset SLE, also in keeping with other studies.^9,32^

The mortality findings were highly significant. We found that the entire cohort had an increased SMR, although the magnitude of difference was far more striking in the JSLE population, with an SMR amongst children under 10 of 87 (CI 27 to 292). The SMR remained increased throughout the age groups, although the magnitude diminished with increasing age. Our group has previously reported a high SMR (with wide CIs) in the UCLH cohort.^13^ This updated analysis has utilized a larger sample size. Our data highlight the significantly increased risk when standardized against the background population which is most pronounced in the youngest population. These data are consistent both with our own group’s previous findings and also with other studies from North America.^34^ A study from Washington published a life-type survival curve which showed no difference over five years between the groups, but the expectation would have been that few if any adolescents should have died. This study did not calculate SMRs.^34^ The trans-American Lupus in Minorities: Nature versus Nurture (LUMINA) trial published mortality figures within their group, by age. They found high numbers of deaths in patients of African American descent in the JSLE group. They reported 11 deaths (six adolescent and five adults) out of a total of 79 patients. These results are far in excess of rates expected.^12^

Our results find that survival has improved over the decades within the cohort. This is consistent with existing publications that report in the adolescent population 10-year survival has improved from 78% in the 1970s to 94% to 100% in 2000s. This may reflect patients being cared for in dedicated units, or increased recognition of the disease and hence earlier diagnosis or milder cases being identified and referred. Groups studying children not under specialist care continue to report higher risk, which appears to relate to socioeconomic status.^12^

Our study has some limitations. We compared two distinct cohorts, which required trimming of datasets to the common information collected by both groups. We are aware of potential selection bias at UCLH, which is a tertiary referral centre, which may skew our adult results to suggest serious manifestations are commoner than if we captured the whole SLE population. Therefore, results indicating higher rates in children (e.g. lupus nephritis) may in reality be higher yet, and a lack of difference (e.g. NPSLE) may miss a real difference. The two cohorts had significantly different median lengths of follow-up, thus definite conclusions of long-term damage were beyond the scope of this paper. We are unable to comment on likely differences in cardiovascular complications between the two groups, as the median ‘event’ occurred at 10 years in the adult cohort, which is longer than the current follow-up of the JSLE cohort.

This study significantly contributes to the current knowledge of SLE phenotypes. It is the first large cohort to look at SLE via the whole age spectrum. The reasons why a phenotype differs depending on age of onset remains a matter of conjecture. SLE is a multifactorial condition and those presenting at a younger age may be more likely to have more genetic contributions and differences in hormone levels, such as oestrogen.^5^ These data confirm an aggressive phenotype of disease in patients with onset of SLE in childhood and adolescence and supports the need for intensive follow-up and therapy in this population.

## Key messages

1. Systemic lupus erythematosus (SLE) is a severe chronic disease that may present at any age and in either gender. Clinical manifestations are similar at all ages but incidence and severity differ.
2. SLE confers an increased standardized mortality ratio, which is particularly pronounced in younger patients.
3. Mature-onset SLE may have a more benign phenotype but with more arthritis.
